# Pleuritis with pleural effusion due to a Bacillus megaterium infection

**DOI:** 10.1002/rcr2.381

**Published:** 2018-11-28

**Authors:** Ernesto Crisafulli, Ilaria Aredano, Ilaria Valzano, Barbara Burgazzi, Francesco Andrani, Alfredo Chetta

**Affiliations:** ^1^ Respiratory Disease and Lung Function Unit, Department of Medicine and Surgery University of Parma Parma Italy

**Keywords:** Bacillus megaterium infection, pleural effusion, pleuritic

## Abstract

Pleural effusions may be related to pleuro‐pulmonary or systemic disorders, including malignancy. Thoracentesis and thoracoscopy may be useful to diagnosis. In some cases, the diagnosis may be difficult and uncommon. We report the case of a hospitalized male for a pleuritis, with pleural effusion caused by a Bacillus megaterium infection, a Gram‐positive, aerobic, spore‐forming, and rod‐shaped bacterium. To our knowledge, our case report is the first evidence of pleuritis due to B. megaterium. In the literature, up to now, B. megaterium infection has only been reported as involving the eye, skin, and brain.

## Introduction

Pleural effusions may be a clinical manifestation of pleuro‐pulmonary or systemic disorders, including malignancy. Dyspnoea and pleuritic chest pain are common clinical manifestations of pleural effusions, although they could sometimes be asymptomatic. Diagnostic techniques identify the cause of about 70% of pleural effusions; however, the aetiology remains uncertain in up to 25% of the patients 1. The first approach includes an accurate anamnesis investigating comorbidities, previous infections (including pulmonary tuberculosis), occupational exposure, and pharmacological anamnesis. A chest computerized tomography (CT) scan should be performed in all patients with undiagnosed pleural effusion; it can identify any irregularities of the pleura and the adjacent parenchyma, and it identifies optimal sites for biopsies. Thoracentesis allows a first evaluation of the physical and chemical characteristics of the pleural fluid. Medical thoracoscopy permits the direct visualization of the pleura and targeted biopsies of the parietal pleura. When biopsies are satisfactory, histological analysis is often necessary to achieve a diagnosis. Despite all, in some cases, a differential diagnosis for pleural effusions may be considered a challenge.

## Case Report

A 77‐year‐old‐man was admitted to our respiratory department for an organized, unilateral pleural effusion. He reported a severe former smoking habit (150 pack/years). He worked mainly as a tailor and, for some years, as a metalworker; he is currently retired, and he spends his time in his garden. His medical history demonstrated that he suffered from arterial hypertension, treated with 10 mg of Olmesartan once daily. He underwent partial gastrectomy some years before for a peptic ulcer. In the month before the admission, he reported a stroke, which led to slight dysarthria; because of this, he is currently on therapy of 300 mg Aspirin once daily. He denied any clinical history of ischaemic heart disease, heart failure, or diabetes. No apparent sources of asbestos exposure were known.

**Figure 1:**
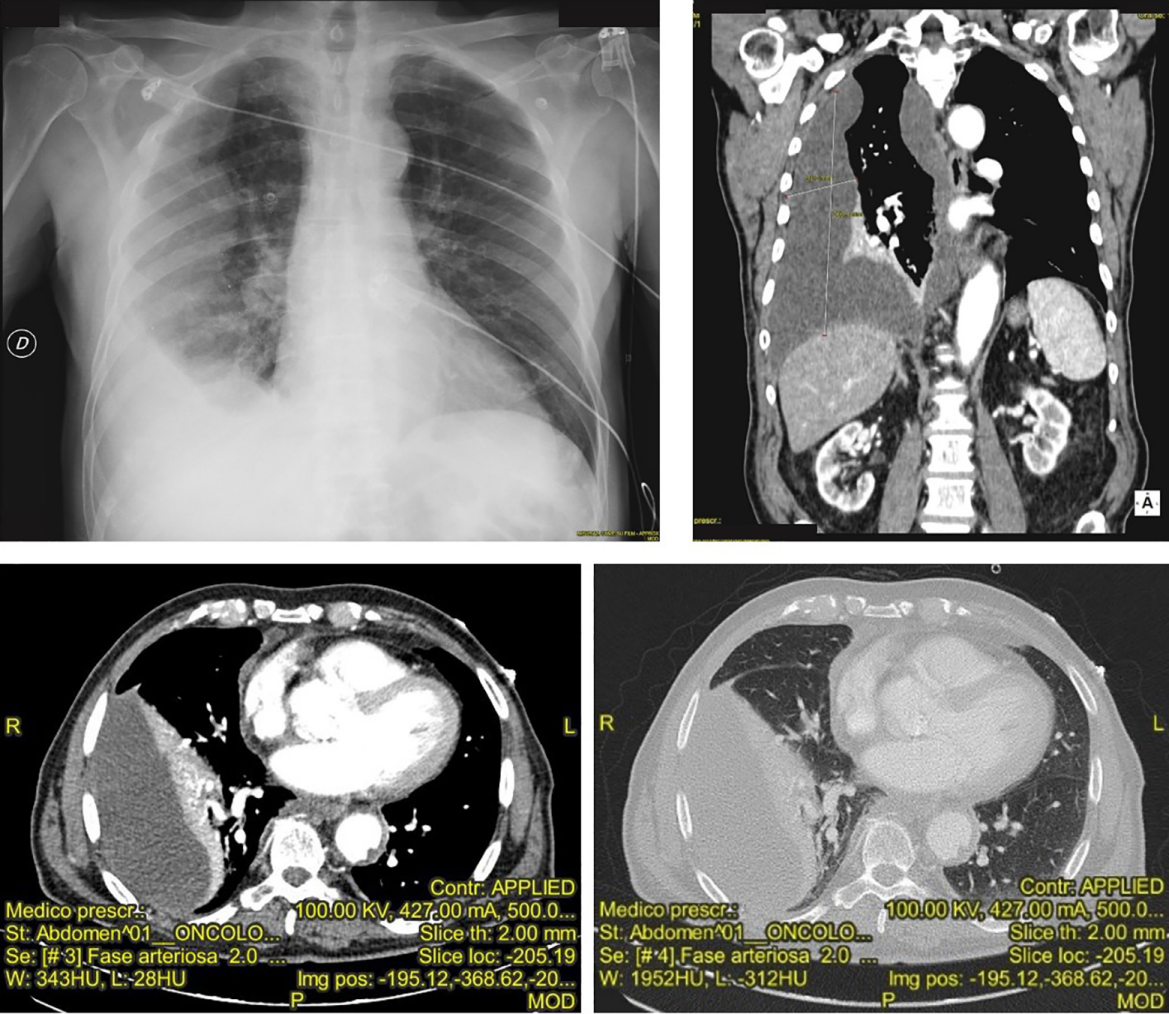
Chest X‐ray and computerized tomography showing a right, organized pleural effusion.

In the month of April 2018, he came to the emergency department of our hospital for acute dyspnoea and tachyarrhythmia (166 beats/min) with a normal arterial pressure (120/80 mmHg). He had a partial arterial oxygen pressure (PaO_2_) of 57 mmHg with normal partial arterial carbon dioxide pressure (PaCO_2_) and pH. The patient also complained of exertional dyspnoea from a few months. He denied fever, cough, and chest pain in the previous months. Blood tests showed macrocytic anaemia (haemoglobin 11 g/dL, mean cell volume 104 fL), a slight value of inflammatory response (leucocytes 10.5 × 10^9^/L with 80% neutrophils, C‐reactive protein 28.70 mg/L). The electrocardiograph showed a paroxysmal atrial fibrillation, which was treated with amiodarone and oral anticoagulation therapy. The chest X‐ray showed a unilateral, organized pleural effusion (Fig. 1). A chest CT scan showed a right, organized pleural effusion and a thickening of the right parietal and mediastinal pleura, suggestive of malignant pleural disease, without mediastinal lymph node involvement but with a compressive atelectasis of the adjacent lung parenchyma (Fig. 1).

On the suspicion of pleural mesothelioma or a pleural localization of a lymphoproliferative disease, the patient underwent medical thoracoscopy, in which multiple biopsies were performed and a pleural drainage was performed, with a removal of 850 mL of pleural fluid. Physical examination of the pleural fluid showed a corpusculated, orange‐coloured liquid with many cords of fibrin; chemical analysis showed an acid pH (7.18) and lactate 9.9 mmol/L, with pleural fluid lactate dehydrogenase (LDH) of 7849 IU/L and total proteins of 5.7 g/dL. Light’s criteria were positive for an exudative pleural effusion. Cytology showed a relevant inflammatory pattern, mainly composed of neutrophils (60.9% of total) and lymphocytes (36.5% of total); no neoplastic cells were described. The research of mycobacteria tuberculosis through in vitro culture was negative. A peripheral lymphocyte subset typing method by cytofluorometry excluded lymphoid malignancies.

**Figure 2:**
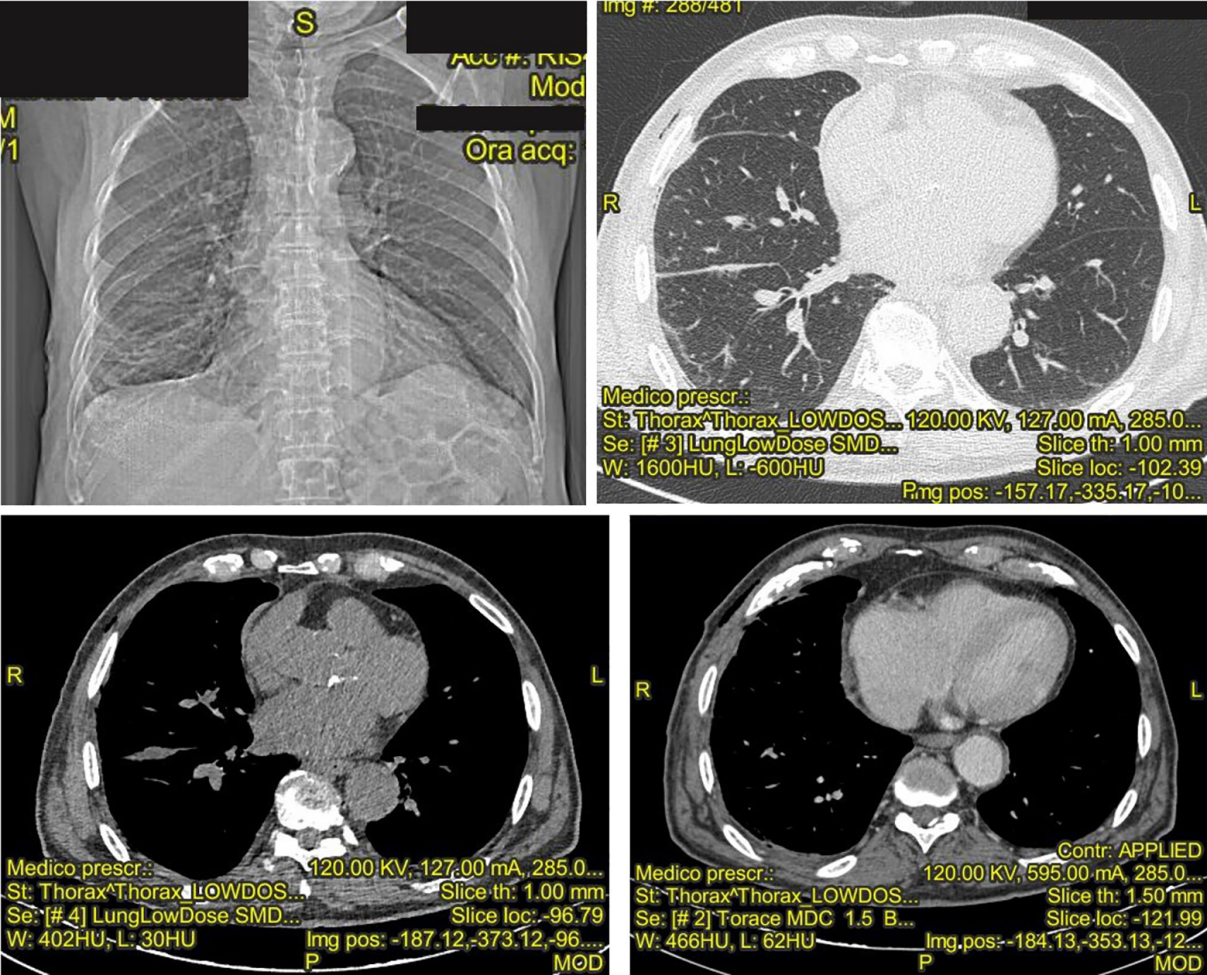
Chest X‐ray and computerized tomography showing the resolution of pleuritis and pleural effusion after antibiotic therapy sensible to Bacillus megaterium.

Histological exam performed on biopsies and pleural fluid showed a “purulent pleuritis” with evidence of *Bacillus megaterium* infection, which was sensible to all antibiotics tested, except for clindamycin. Therapy with meropenem of 3 g/day and levofloxacin of 500 mg/day was started, with benefits. A chest CT scan after two weeks of the antibiotic therapy showed a significant improvement (Fig. 2) with normality of blood exams. The patient was no longer dyspnoeic, without a need for oxygen. The patient achieved completed resolution.

## Discussion

*B. megaterium* is a Gram‐positive, aerobic, spore‐forming, and rod‐shaped bacterium, found in diverse habitats. Recently, this bacterium has been used in biotechnology as a source of recombinant protein 2.

The infections caused by *B. megaterium* are rare, and to our knowledge, it has never been reported as the cause of a pleuritis with a pleural effusion. In literature, only three cases of infection are described. In 2006, Ramos‐Esteban J.C. and colleagues reported a case of a 23‐year‐old man who developed delayed onset lamellar keratitis caused by this infection two weeks after laser‐assisted in situ keratomileusis (LASIK) for correction of myopia 3. In this case, the bacteria entered the eye through the surgical wound on the cornea; in our patients, no surgical wound or abrasion of the skin was present in the previous period the admission. In 2011, Duncan and Smith reported the case of a 25‐year‐old woman who presented with a primary cutaneous infection caused by *B. megaterium*, probably acquired by micro abrasions on the skin 4. Finally, in 2015, Guo et al. reported the case of a 50‐year‐old woman who developed a brain abscess related to an infection with *B. megaterium* 5. In this case, the method of entrance was not clear, as in our case report. The ability to cause a purulent infection in an anaerobic setting is common to all cases.

*B. megaterium* is typically assumed to be non‐pathogenic or, at least, of very low virulence. In our patient, the pleural localization of *B. megaterium* has caused a subacute infection that has been likely acquired by inhaling the bacterium in the environment, probably related to his exposure to soil in his garden. Although the broad spectrum of antibiotic sensitivity has given us so many therapeutic possibilities, we should re‐evaluate the pathogenic capacity of these Bacillus species.

## Disclosure Statement

Appropriate written informed consent was obtained for publication of this case report and accompanying images.
